# Phylogeny-guided curation reveals widespread misannotation of Asgard archaeal 16S rRNA gene sequences in public databases

**DOI:** 10.1093/ismeco/ycag253

**Published:** 2026-09-04

**Authors:** Agathe Struillou, Philippe Deschamps, David Moreira, Purificación López-García

**Affiliations:** Ecologie Société Evolution, CNRS, Université Paris-Saclay, AgroParisTech, 91190 Gif-sur-Yvette, France; Ecologie Société Evolution, CNRS, Université Paris-Saclay, AgroParisTech, 91190 Gif-sur-Yvette, France; Ecologie Société Evolution, CNRS, Université Paris-Saclay, AgroParisTech, 91190 Gif-sur-Yvette, France; Ecologie Société Evolution, CNRS, Université Paris-Saclay, AgroParisTech, 91190 Gif-sur-Yvette, France

**Keywords:** Asgardarchaeota, 16S rRNA, taxonomic annotation, molecular phylogeny, sequence databases, metagenome-assembled genomes

## Abstract

Accurate taxonomic assignment of 16S rRNA gene sequences is essential for the reliable interpretation of microbial community studies based on amplicon sequence data. Yet, it critically depends on the reliability of reference databases such as the Genome Taxonomy Database (GTDB) and the SILVA ribosomal RNA database. Here, we evaluate the consistency of taxonomic annotations within the Asgardarchaeota phylum, a lineage of major evolutionary and ecological interest. Using a phylogenetically curated set of GTDB-derived 16S rRNA gene sequences, we show that most of the affiliations of these sequences were consistent with the phylogenomic placement of their corresponding metagenome-assembled genomes (MAGs), although a small fraction of them exhibited clear inconsistencies likely resulting from erroneous binning to MAGs. In contrast, phylogenetic analyses of SILVA-derived 16S rRNA gene sequences including curated reference sequences revealed widespread taxonomic misannotation and/or limited resolution of taxon assignment. Specifically, many sequences annotated as Odinarchaeales robustly clustered within Lokiarchaeia, Heimdallarchaeia, Hermodarchaeia, or Sifarchaeia, leading to an artificial inflation of Odinarchaeales assignments and potentially biased ecological interpretations. To mitigate these issues, we constructed a curated reference dataset of Asgardarchaeota 16S rRNA gene sequences and generated phylogenetically validated taxonomic assignments across clustered entries, providing a resource for improved classification of environmental sequences. Our results demonstrate that widely used reference databases can contain systematic annotation errors that propagate across studies and distort ecological inference. Although illustrated using Asgard archaea, these limitations are likely pervasive across understudied microbial diversity, highlighting the need for routine phylogenetic validation and systematic curation of reference datasets.

Accurate taxonomic assignment of microbial sequences is a cornerstone of modern microbial ecology, as it underpins analyses of diversity, community structure, and ecological function [1–3]. Over the years, widely used reference databases, such as the SILVA ribosomal RNA database and the Genome Taxonomy Database (GTDB), have become essential sources for assigning taxonomy to, respectively, 16S rRNA and protein-coding gene sequences [4–7]. However, their widespread use also implies that systematic annotation errors may propagate across numerous ecological studies.

An underappreciated limitation of these resources relates to inconsistent or imprecise taxonomic assignments [8, 9], which complicate ecological and evolutionary interpretations. SILVA integrates a broad diversity of ribosomal RNA sequences but also contains misannotated entries or annotations limited to high taxonomic ranks, with many lineages remaining unresolved (*incertae sedis*), limiting fine-scale ecological interpretation. In GTDB, many metagenome-assembled genomes (MAGs) lack rRNA genes but, when they are present, it is possible to link the more robust phylogenomic (multigene) assignment of MAGs to their associated 16S rRNA genes. This is especially useful for lineages lacking cultured representatives. However, 16S rRNA gene sequences may occasionally be incorrectly linked to MAGs due to misassembly, spurious binning, or other errors, leading to mismatches between rRNA-based and multigene-based taxonomy.

These limitations may be particularly pronounced for taxa with none or few uncultured members, such as the archaeal phylum Asgardarchaeota, a lineage of growing evolutionary interest due to its relevance to eukaryogenesis [10–12]. The rapid expansion of asgardarchaeal sequences in public databases amplifies this risk. Here, we used Asgard archaea as a case study to evaluate the consistency of taxonomic assignments in GTDB and SILVA. We reconstructed a curated dataset of 16S rRNA gene sequences based on detailed phylogenetic analyses and assessed the extent to which GTDB- and SILVA-based annotations reflect phylogenetic structure. Beyond identifying inconsistencies, we provide a phylogeny-guided framework for improving taxonomic resolution through systematic curation.

We first retrieved 16S rRNA gene sequences associated with Asgard archaeal genomes and MAGs from the GTDB release r226 and retained only high-quality 1200–1900 bp-long sequences without undetermined nucleotides, located on contigs longer than 5 kb. For genomes containing multiple 16S rRNA gene copies, the first occurrence meeting those criteria was selected. We aligned these GTDB asgardarchaeal 16S rRNA gene sequences with 107 representative sequences spanning the diversity of other archaeal clades (Euryarchaeota, DPANN, and TACK [13]) using MAFFT [14] with the accurate L-INS-i options (-localpair --maxiterate 1000), and inferred a maximum-likelihood phylogenetic tree with IQ-TREE [15] (v3.0.1) with the GTR+G4 substitution model. Branch support was assessed using 1000 ultrafast bootstrap replicates (-B 1000) [16] and 1000 SH-aLRT (Shimodaira–Hasegawa approximate likelihood ratio test) replicates (--alrt 1000) [17]. The phylogenetic tree was iteratively refined to improve the representation of under-sampled Asgard archaeal lineages (e.g. Hodarchaeales, Helarchaeales and Sigynarchaeaceae, renamed Sigyniarchaeaceae in GTDB release r232, April 2026): following reconstruction of an initial phylogenetic tree using high-quality GTDB sequences, additional GTDB-derived 16S rRNA gene sequences assigned to poorly represented clades were incorporated, even if they were not fully satisfying the initial quality criteria, before a new round of phylogenetic tree inference. Sequences that robustly clustered within their expected lineages were retained to improve clade representation, whereas redundant sequences or sequences robustly placed within clades inconsistent with their assigned taxonomy were discarded. This iterative curation procedure improved the sampling of underrepresented lineages while maintaining a phylogenetically coherent reference backbone. This final curated reference comprised 72 high-confidence asgardarchaeal 16S rRNA gene sequences.

To ascertain the correct affiliation of additional 16S rRNA gene sequences assigned to Asgardarchaeota in public databases, we combined the 72 curated GTDB-derived reference sequences with 219 additional non-reference GTDB sequences and 4499 SILVA sequences to build a final curated dataset (provided as supplementary material; Supplementary Tables S1–S3; Supplementary Data S1–S4). To construct this dataset, we clustered all these sequences at 99% identity using CD-HIT [18], which yielded 830 clusters, each of them containing a representative sequence. The representative sequences originated from either GTDB (*n* = 168) or SILVA (*n* = 662), although individual clusters could contain sequences from both sources. Two cultured Asgard archaeal 16S rRNA gene sequences (*Prometheoarchaeum syntrophicum* [19] and *Lokiarchaeum ossiferum* [20]) were subsequently added to the phylogenetic dataset (Supplementary Fig. S1). To assess the taxonomic homogeneity of CD-HIT clusters, we examined the taxonomic assignments of the sequences they contained at the class, order and family levels, when including and excluding the *incertae sedis* category. Of the 830 clusters, 598 (72%) were singletons and were therefore excluded from the taxonomic composition analyses. The remaining 235 multi-sequence clusters were largely homogeneous across the three taxonomic ranks examined (Supplementary Fig. S2; Supplementary Tables S4–S11).

After aligning cluster representative sequences for this extended dataset, we inferred a new phylogenetic tree and assessed the congruence between the taxonomic annotation of the sequences and their phylogenetic placement within well-supported monophyletic groups consistent with the GTDB taxonomy (Supplementary Fig. S1). We identified misannotated sequences robustly branching within clearly supported clades and reassigned their taxonomic affiliation accordingly, propagating the corrected taxonomy to all sequences within each (≥99% identity) cluster.

Our analyses were performed using the GTDB release r226. A more recent release (r232) introduced updates to Asgardarchaeota taxonomy, including the reclassification of MK-D1 into *Promethearchaeaceae* and the renaming of *Sigynarchaeales|Sigynarchaeaceae* to *Sigyniarchaeales|Sigyniarchaeaceae*. These nomenclatural changes, however, do not affect the purpose, results and conclusions of this study.

Most 16S rRNA gene sequences from GTDB MAGs clustered within well-supported, taxonomically coherent clades consistent with their phylogenomic-based assignments, i.e. 16S rRNA gene sequences are generally correctly associated with their MAGs. Only a small number of sequences robustly branched in phylogenetic positions incompatible with those of their associated MAGs. For example, six sequences associated with asgardarchaeal MAGs clustered within Euryarchaeota, DPANN or TACK lineages (e.g. GB_GCA_030587365.2, GB_GCA_038826975.1, GB_GCA_937877725.1, GB_GCA_038825615.1; Supplementary Fig. S1), suggesting incorrect binning or gene-to-genome association [21, 22]. These misassigned 16S rRNA gene sequences should be excluded from the corresponding MAGs to avoid further errors. In some cases, wrong associations likely originated from some level of MAG contamination resulting in multiple 16S rRNA gene copies. For example, the MAG GB_GCA_016839605.1, annotated as Thorarchaeia, contained a 16S rRNA gene copy branching within Thorarchaeia and a second copy branching with maximal support within Hermodarchaeia (Supplementary Fig. S1).

In contrast, molecular phylogenetic analyses of SILVA-derived 16S rRNA gene representative cluster sequences and curated reference sequences revealed substantial inconsistencies. Particularly, out of the 105 cluster sequences annotated as Odinarchaeales in the SILVA database, 49 robustly branched within the Heimdallarchaeia family JABLTI01, and 29 within the Lokiarchaeia family MK-D1 (Promethearchaeaceae in GTDB r232). Other sequences also annotated as Odinarchaeales branched within other Asgard lineages, including Sifarchaeia (6, Borrarchaeales|Borrarchaeaceae), Hermodarchaeia (4, Hermodarchaeales|Hermodarchaeaceae), Heimdallarchaeia (4, Hodarchaeales), and Lokiarchaeia (3, Helarchaeales) (Figs. 1A and B; Supplementary Fig. S1). In fact, only 3 out of the 105 “Odinarchaeales” representative cluster sequences were phylogenetically confirmed. Moreover, these annotations were internally inconsistent from a taxonomic hierarchy standpoint. Thus, in the SILVA taxonomy, these sequences were simultaneously assigned to the class Lokiarchaeia and the order Odinarchaeales (e.g. Fig. 1B, Supplementary Fig. S1) although, based on multi-gene phylogenomic analysis [23], Odinarchaeales belongs to the class Odinarchaeia in the GTDB taxonomy. The pronounced SILVA bias towards assignment of (diverse) asgardarchaeal sequences as Odinarchaeales artificially inflates the apparent diversity and ecological distribution of this order which, in reality, seems exclusively composed of thermophilic members, thriving in high-temperature ecosystems [11, 24]. As a consequence, ecological patterns inferred from SILVA-based taxonomy may reflect database artefacts rather than true biological distributions. Such systematic misannotation is likely to get amplified, as incorrectly annotated sequences are used to classify new ones, reinforcing taxonomic biases and potentially distorting downstream interpretations.

**Figure 1:**
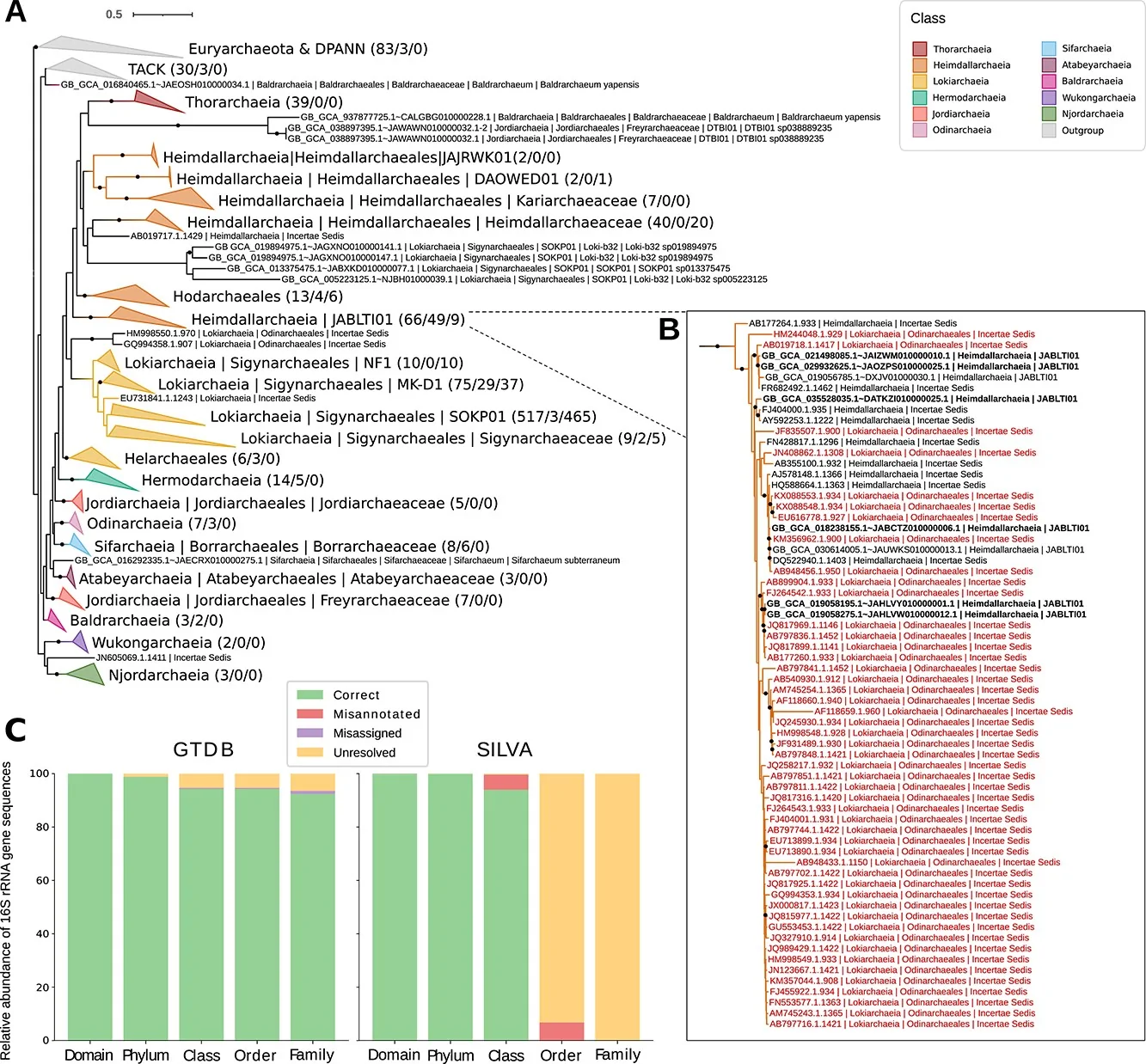
Phylogenetic analysis of GTDB and SILVA Asgardarchaeota 16S rRNA gene sequences highlighting misannotation problems in public databases. (A) Maximum-likelihood phylogenetic tree of Asgardarchaeota 16S rRNA gene sequences. Nodes with ultrafast bootstrap support ≥99 are indicated by black dots. Clades were collapsed (original tree in Supplementary Fig. S1); the total number of sequences in the clade is indicated in parentheses, followed by the number of misassigned asgardarchaeal sequences and that of unresolved sequences (*incertae sedis*). (B) The inlet shows an expanded view of the heimdallarchaeal JABLTI01 order, showing an example of sequences incorrectly annotated in SILVA as Lokiarchaeia/Odinarchaeales (in red). Reference sequences from the curated GTDB backbone are shown in bold. Scale bar indicates the number of nucleotide substitutions per site. (C) Quality of asgardarchaeal 16S rRNA gene sequence classification across taxonomic ranks for GTDB and SILVA sequences. Sequences were classified as correctly annotated, unresolved (*incertae sedis*), misannotated (SILVA) or misassigned (GDTB) based on phylogenetic placement in the curated backbone tree.

Considering our full dataset of 16S rRNA gene sequences, the GTDB-based annotations remained highly consistent across taxonomic ranks, retaining high accuracy even at the family level (92.42%; 269/291 GTDB sequences), with only minimal misassignment (1.14%; 3/291 GTDB sequences) and limited proportion of unresolved sequences (6.44%; 19/291 GTDB sequences) (Fig. 1B). In contrast, SILVA showed a sharp non-linear decline in resolution at finer ranks. While accuracy remained high at domain and phylum levels (99.98%; 4498/4499 sequences), correct assignments dropped to 0.16% at order level (7/4499 SILVA sequences) and 0.04% at family level. Most sequences were classified as unresolved at these ranks (*incertae sedis*) at order (93.22%; 4194/4499 SILVA sequences) and family (99.96%; 4497/4499 SILVA sequences) levels, respectively. While in GTDB inconsistencies arise from MAG redundancy (diverse gene copies binned to the same MAGs) or otherwise incorrect binning, in SILVA they are caused by both, actual misannotation (e.g. sequences wrongly assigned to Odinarchaeales) and lack of resolution, the latter being a less problematic phenomenon. Most apparent taxonomic ambiguity in SILVA reflects limitations in sequence annotation rather than intrinsic phylogenetic uncertainty, as our phylogeny-guided curation resolved most ambiguous cases, recovering up to 99.83% and 99.67% of assignments at, respectively, order and family levels (Fig. 2A).

**Figure 2:**
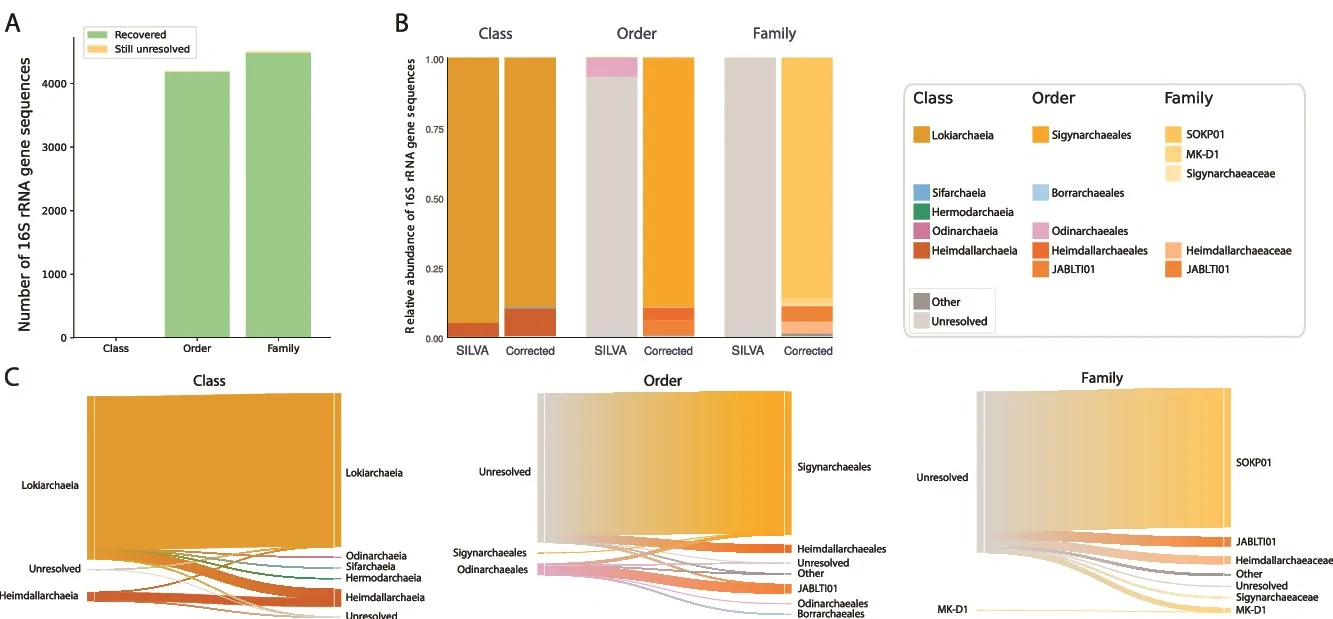
Recovery of unresolved asgardarchaeal taxa and major taxonomic reassignments through 16S rRNA gene phylogeny-guided curation. (A) Number of SILVA *incertae sedis* sequences correctly assigned to specific taxa. (B) Proportion of 16S rRNA gene sequence taxonomic annotation in SILVA before and after phylogenetic curation (corrected). Only the 6 most abundant taxa are shown individually, less represented groups are grouped under the category “Other.” *Incertae sedis* are grouped as “Unresolved.” (C) Sankey diagrams showing taxonomic transitions following curation. Flows represent redistribution of SILVA-derived sequences from original to phylogenetically corrected assignments at the class, order and family levels. For each plot, the left side shows original SILVA taxonomic annotations and the right side the corrected and propagated taxonomy inferred from phylogenetic placement. Flow widths are proportional to sequence counts, and colors represent the transition between original and corrected taxonomic assignments using a source-to-target gradient. Across all plots, only the most represented taxa are displayed individually (corresponding to the top six most abundant taxa retained at each rank), while less abundant groups are grouped under the category “Other.”

At the class level, the most important corrections involved the reassignment of 234/4499 (5.2%) SILVA sequences initially annotated as Lokiarchaeia to Heimdallarchaeia. At the order level, 233/4499 (5.2%) SILVA sequences annotated as Odinarchaeales were reassigned to the Heimdallarchaeia JABLTI01, while 3967/4499 (88.2%) previously unresolved SILVA sequences could be resolved as Sigynarchaeales (Sigyniarchaeales in GTDB r232). At the family level, phylogenetic curation primarily recovered previously unresolved diversity, with 3880/4499 (86.2%) SILVA sequences assigned to SOKP01 (Promethearchaeaceae in GTDB r232) (Figs. 2B and C; Supplementary Tables S12–S14). This indicates that Lokiarchaeia represents the dominant Asgard lineage in SILVA, with 4273/4499 sequences (95%) initially assigned to this class. After phylogenetic curation, 4020/4499 sequences remained assigned to Lokiarchaeia (89.40%). These shifts reveal that database-dependent biases can strongly influence the analysis of the composition of microbial communities, especially at ecologically relevant taxonomic scales, such as orders and families. While they seem to be widely distributed and abundant in metagenomes from many ecosystems [11, 25], it is surprising that Thorarchaeia are virtually absent from rRNA databases (0/4499 16S rRNA gene sequences in the SILVA dataset analyzed here). This confirms and extends predicted primer biases against this clade [26], recently verified experimentally [27].

Despite the aforementioned annotation problems, most inconsistencies in SILVA can be resolved through phylogeny-guided curation. By combining clustering at 99% sequence identity with phylogenetic placement in a curated backbone tree and propagating validated assignments to all cluster members, near-complete resolution can be achieved at ecologically relevant ranks. Our final curated dataset (Supplementary Tables S1–S3; Supplementary Data S1 and S2) can be used to train automatic 16S rRNA classifiers and provides a scalable framework applicable beyond Asgard archaea.

Although we focused on Asgard archaea, as a relevant test case due to their rapid taxonomic expansion and ecological importance, the issues highlighted here reflect broader limitations in microbial sequence reference databases. By explicitly documenting inconsistencies, we aim to raise awareness within the microbial ecology community. We argue that careful phylogenetically informed validation of taxonomic assignments should become a standard practice, particularly when investigating poorly resolved or rapidly expanding lineages. Integration of phylogeny-guided validation into curation pipelines would help prevent propagation of systematic annotation errors and improve future taxonomic frameworks.

## Supplementary Material

Supplementary_material_ycag253

## Data Availability

Sequence data used in this work are publicly available in GenBank (https://www.ncbi.nlm.nih.gov/genbank/), SILVA (https://www.arb-silva.de/) and GTDB (https://gtdb.ecogenomic.org/) databases, and/or uploaded as Supplementary Data S1–S4 files.
