# Supplementary material for: Phylogeny-guided curation reveals widespread misannotation of Asgard archaeal 16S rRNA gene sequences in public databases
Source: ISME Commun. 2026 Sep 4;6(1):ycag253. doi: 10.1093/ismeco/ycag253 (PMC13617573; doi:10.1093/ismeco/ycag253)
Supplement: Supplementary file 1 [file supplementary_material_ycag253.zip › Supplementary_Figures_R1_ycag253.pdf]

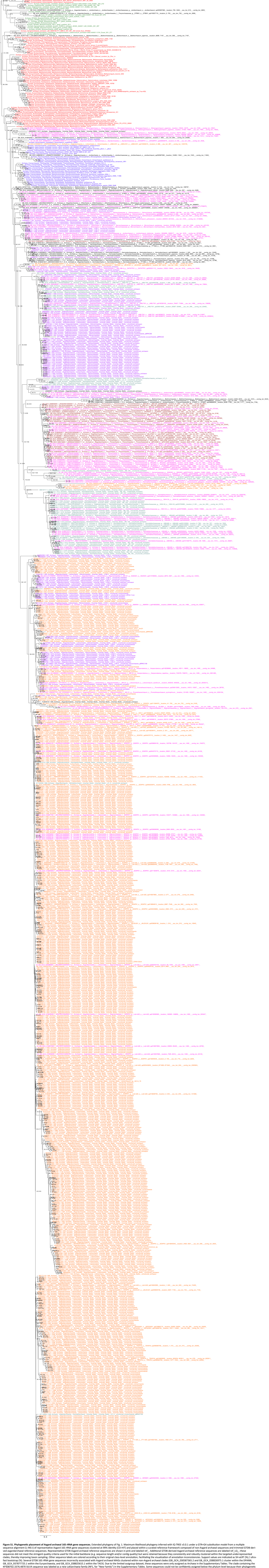

Figure S1. Phylogenetic placement of Asgard archaeal 16S rRNA gene sequences. Extended phylogeny of Fig. 1. Maximum-likelihood phylogeny inferred with IQ-TREE v3.0.1 under a GTR+G4 substitution model from a multiple sequence alignment (L-INS-i) of representative Asgard 16S rRNA gene sequences clustered at 99% identity (CD-HIT) and placed within a curated reference framework composed of non-Asgard archaeal sequences and trimmed GTDB-derived asgardarchaeal reference sequences. Representative GTDB Asgard archaeal reference sequences are shown in pink and labeled ref_. Additional GTDB-derived Asgard archaeal reference sequences are labeled ref_LQ_; these sequences did not meet the stringent quality criteria used for the initial backbone (e.g. sequence length and/or contig quality) but were retained because they consistently and robustly clustered within the targeted underrepresented clades, thereby improving taxon sampling. Other sequence labels are colored according to their original class-level annotation, facilitating the visualization of annotation inconsistencies. Support values are indicated as SH-aLRT (%) / ultrafast bootstrap (%). Several GTDB 16S rRNA gene sequences incorrectly associated with Asgard archaeal MAGs clustered within non-Asgard archaeal clades (GB_GCA_030587365.2 and GB_GCA_038826975.1 cluster within the DPANN, GB_GCA_937877725.1 within the Euryarchaeota, and GB_GCA_038825615.1 within the TACK). Due to the limited outgroup dataset, these sequences were only assigned as Archaea in the Supplementary Tables. The clade containing the KP308702.3376.4870 SILVA sequence was labeled as a new Lokiarchaeia family (NF1, for "New Family") in the Supplementary Tables. Some sequences could not be confidently assigned below the phylum level because their phylogenetic position remained ambiguous. Accordingly, GB_GCA_937877725.1, duplicated sequences GB_GCA_038897395.1, GB_GCA_005223125.1, GB_GCA_013375475.1, duplicated sequences GB_GCA_019894975.1, GQ994358.1.907 and HM998550.1.970 were conservatively assigned only to the phylum Asgardarchaeota in the Supplementary Tables.

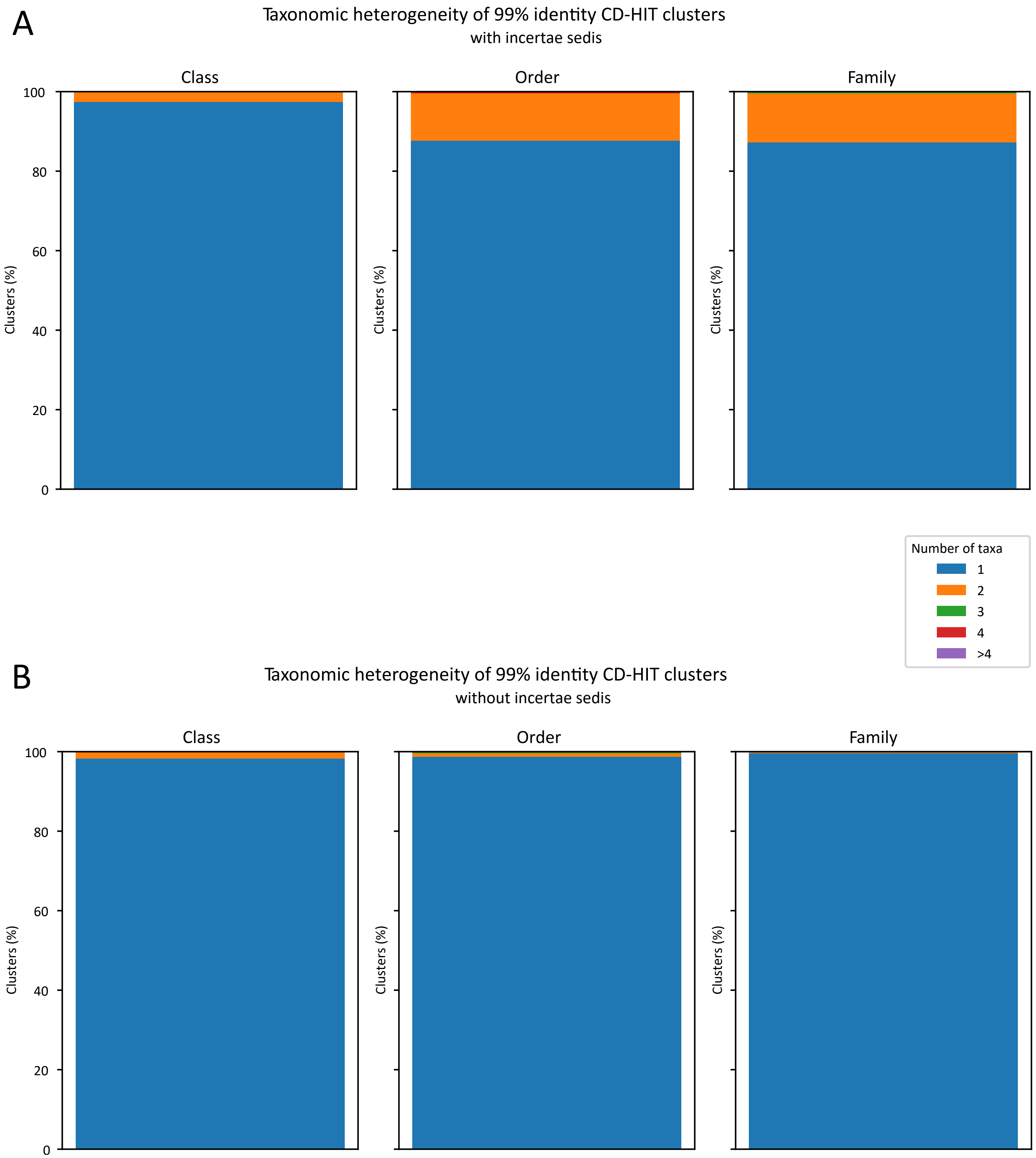

Figure S2. Taxonomic heterogeneity of 99% identity CD-HIT clusters at different taxonomic ranks (class, order, family). Only informative clusters containing at least two sequences were considered (598 singletons (72%); 235 multi-sequence clusters). Stacked bars represent the proportion of the 235 multi-sequence clusters containing one to more than four taxonomic categories at each taxonomic rank. (A) Taxonomic heterogeneity including *incertae sedis* assignments. *Incertae sedis* classifications were considered as distinct taxonomic categories when assessing cluster composition. More than 97% of clusters showed a single class-level assignment and more than 87% showed a single order- or family-level assignment. (B) Taxonomic heterogeneity after excluding *incertae sedis* assignments. *Incertae sedis* categories were ignored when evaluating cluster composition; clusters composed exclusively of *incertae sedis* sequences were considered homogeneous. More than 98% of clusters were homogeneous across all three taxonomic ranks.
